# Roles of Rho GTPases in Intracellular Transport and Cellular Transformation

**DOI:** 10.3390/ijms14047089

**Published:** 2013-03-28

**Authors:** Xiaojuan Chi, Song Wang, Yifan Huang, Mark Stamnes, Ji-Long Chen

**Affiliations:** 1 College of Animal Science, Fujian Agriculture and Forestry University, Fuzhou 350002, China; E-Mails: chixiaojuan88@126.com (X.C.); zjhyfang@163.com (Y.H.); 2 CAS Key Laboratory of Pathogenic Microbiology and Immunology, Institute of Microbiology, Chinese Academy of Sciences (CAS), Beijing 100101, China; E-Mail: wscookie@163.com; 3 Department of Molecular Physiology and Biophysics, Roy J. and Lucille A. Carver College of Medicine, The University of Iowa, Iowa City, IA 52242, USA; E-Mail: mark-stamnes@uiowa.edu

**Keywords:** Rho GTPases, vesicle trafficking, viral transport, cellular transformation, actin cytoskeleton

## Abstract

Rho family GTPases belong to the Ras GTPase superfamily and transduce intracellular signals known to regulate a variety of cellular processes, including cell polarity, morphogenesis, migration, apoptosis, vesicle trafficking, viral transport and cellular transformation. The three best-characterized Rho family members are Cdc42, RhoA and Rac1. Cdc42 regulates endocytosis, the transport between the endoplasmic reticulum and Golgi apparatus, post-Golgi transport and exocytosis. Cdc42 influences trafficking through interaction with Wiskott-Aldrich syndrome protein (N-WASP) and the Arp2/3 complex, leading to changes in actin dynamics. Rac1 mediates endocytic and exocytic vesicle trafficking by interaction with its effectors, PI3kinase, synaptojanin 2, IQGAP1 and phospholipase D1. RhoA participates in the regulation of endocytosis through controlling its downstream target, Rho kinase. Interestingly, these GTPases play important roles at different stages of viral protein and genome transport in infected host cells. Importantly, dysregulation of Cdc42, Rac1 and RhoA leads to numerous disorders, including malignant transformation. In some cases, hyperactivation of Rho GTPases is required for cellular transformation. In this article, we review a number of findings related to Rho GTPase function in intracellular transport and cellular transformation.

## 1. Introduction

The Rho GTPase family, which consists of more than 20 proteins in humans, can be categorized into eight subfamilies according to amino-acid sequence similarities: Rac (Rac1, Rac2, Rac3 and RhoG); Cdc42 (Cdc42, TC10 and TCL); CHP and WRCH1; RhoH; Rho BTB (RhoBTB1, RhoBTB2); Rho (RhoA, RhoB and RhoC); RND (RND1, RND2 and RND3); and Rif (Rif and RhoD) [1]. The members of the four subfamilies (Rac, Cdc42, Rho and Rif) belong to the classical Rho GTPases, while others are parts of the atypical Rho GTPases. Over the past twenty years, Rho GTPases have been implicated in a variety of cellular processes, especially in the regulation of cytoskeletal dynamics [2,3]. Extensive studies have focused on the functional analysis of three important Rho GTPase members: Cdc42, Rac1 and RhoA. Interestingly, recent studies have shown that these Rho GTPases are also involved in cellular trafficking and tumorigenesis.

**Figure 1:**
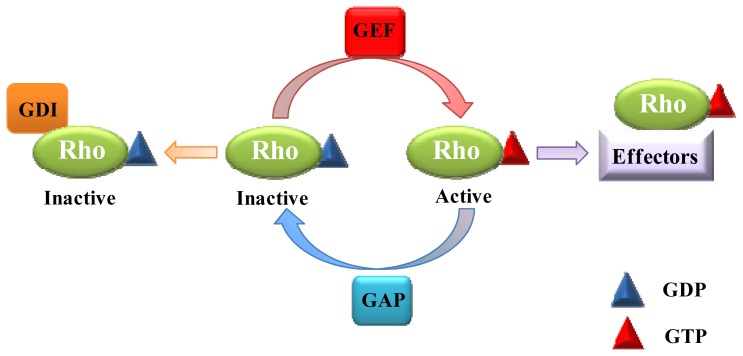
Rho GTPase activity is controlled by guanine nucleotide exchange factor (GEF), GTPase-activating protein (GAP) and guanine nucleotide dissociation inhibitor (GDI). GEF activates Rho GTPases by facilitating the release of GDP and the binding of GTP. GAP inactivates Rho GTPases by promoting hydrolysis of the bound GTP molecules, resulting in their quick change from the GTP-bound form to the GDP-bound form. GDI binds to *C*-terminal prenyl groups on some Rho proteins, maintaining them in the inactive state. Active Rho GTPases act on their downstream effector proteins, stimulating a variety of cellular processes.

Like other GTPases, the classical Rho GTPases cycle between active GTP-bound forms and inactive GDP-bound forms. There are three types of regulatory proteins that control the GTPase cycle: guanine nucleotide exchange factors (GEFs), GTPase-activating proteins (GAPs) and guanine nucleotide dissociation inhibitors (GDIs) [4] (Figure 1). GEFs facilitate the release of GDP and the binding of GTP to activate GTPases. GAPs inactivate Rho GTPases by promoting the intrinsic GTP hydrolyzing activity of Rho proteins, resulting in a GDP-bound form. GDIs can bind to *C*-terminal prenyl groups on some Rho proteins to keep the GTPases in the inactive form [5]. Through this GTPase cycle, Rho proteins, together with their GEFs, GAPs and GDI regulators, function as molecular switches in the regulation of a wide range of cellular processes, including cell polarity, cellular adhesion, vesicle trafficking, migration, neuronal development, morphogenesis, proliferation, differentiation and transformation [6–9].

Normal temporal and spatial regulation of vesicular transport events is crucial to cell proliferation and apoptosis, as well as the maintenance of homeostasis. For instance, inhibition of brefeldin A (BFA)-inhibited GEF2 protein (BIG2), which is important for vesicular transport, decreases cell proliferation and disturbs the intracellular localization of E-cadherin and β-catenin, thereby influencing human cerebral cortical development [10]. Deregulation of vesicular transport can lead to decreased capacitive calcium entry, which in turn results in cell apoptosis [11]. Previous studies have indicated that many Rho GTPases regulate transport pathways, including the endoplasmic reticulum (ER) to the Golgi and endocytic and exocytic transports. In many cases, Rho GTPases play a key role in vesicle trafficking through their ability to regulate the actin cytoskeleton. The Rho GTPases, in an active GTP-bound form, interact with downstream effectors that either directly or indirectly stimulate actin polymerization. Actin plays multiple roles in vesicle trafficking [12–14]. It not only facilitates membrane deformation, cytoskeleton remodeling and the formation of vesicles, but also contributes to vesicle movement and targeting within the cell.

The best-studied Rho family members are Cdc42, Rac1 and RhoA in mammalian cells. Activation of Cdc42 helps control cytoskeletal remodeling, establishment of cell polarity, migration, proliferation and transcription by stimulating a variety of signaling cascades [15,16]. In addition, Cdc42 participates in intracellular trafficking and the regulation of malignant transformation, tumor progression and metastasis [17,18]. Rac1 is ubiquitously expressed and involved in signal pathways that regulate mobility and other processes related to membrane trafficking and cell morphology [19,20]. RhoA facilitates the assembly of contractile actomyosin filaments and is associated with focal adhesion complexes. Recently, RhoA has been found to function in vesicle trafficking, tumor invasion and metastasis [21,22].

Other Rho family members, in addition to Cdc42, Rac1 and RhoA, have also been implicated in vesicle trafficking and cell transformation. TC10 is an important molecule for exocytic processes and interacts with one of the members of the exocyst complex, Exo70, which is a major player in exocytosis and has been identified as an effector of Cdc42 and Rho3 in yeast [23,24]. It can regulate the trafficking of glucose transporter 4 (GLUT4) and cystic fibrosis transmembrane conductance regulator (CFTR) [25,26]. Since insulin-mediated activation of TC10 promotes the exocytic processes of GLUT4, it may associate with the development of type 2 diabetes. In human neutrophils, Rac2 can promote primary granule exocytosis through mediating actin cytoskeletal remodeling [27,28]. RhoD is able to link actin reorganization to endosomal vesicle transport [29]. RhoB not only regulates endocytic trafficking, but also acts as a tumor suppressor. Dominant-negative RhoB (RhoBN19 and RhoBV14) affects postendocytic traffic of ligand-receptor complexes from the basolateral endosomes to apical compartments [30]. Furthermore, the activated form of RhoB can delay the endocytic trafficking of the epidermal growth factor receptor (EGFR) [31]. RhoB has also been reported to inhibit tumor growth, cell migration and invasion [32], but the mechanism by which it suppresses tumor progression and invasion is still unclear. RhoC has been identified for involvement in tumor metastasis [33]. Rac1b, a tumor associated and constitutively active Rac1 splice variant, is upregulated in colorectal tumor cell and can promote transformation of NIH3T3 cells [34,35]. In this review, we focus on the trafficking and cancer-related functions of Cdc42, Rac1 and RhoA in mammalian cells.

## 2. Role of Rho GTPases in Vesicular Transport

### 2.1. Cdc42: A Regulator of Vesicle Trafficking

**Figure 2:**
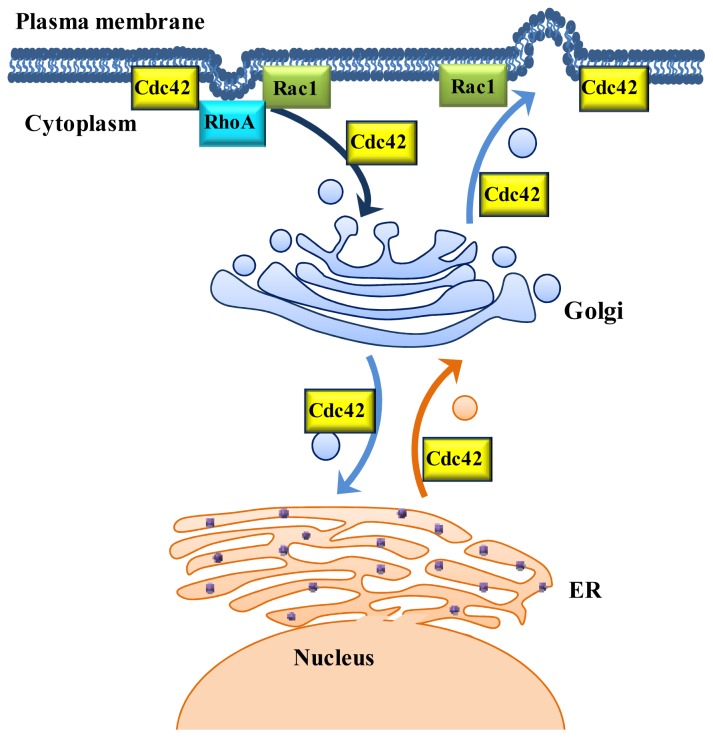
Cdc42, Rac1 and RhoA function in vesicle trafficking. Cdc42 is implicated in endocytosis, trafficking from the cell surface to Golgi region, transport between the endoplasmic reticulum (ER) and Golgi, post-Golgi transport and exocytosis. Rac1 involves in endocytosis and exocytosis, while RhoA mainly takes part in endocytosis.

Cdc42, a highly conserved small GTPase of the Rho family, was first discovered in the budding yeast *Saccharomyces cerevisiae*, due to its involvement in the development of cell polarity [36,37]. Since then, Cdc42 has been found to be a molecular switch that modulates a variety of cellular processes in organisms from yeast to mammals, including regulation of actin cytoskeletal architecture and cell polarity, which have been extensively studied. Several studies have implicated Cdc42 in endocytosis, ER/Golgi interface vesicle trafficking, including anterograde (ER-to-Golgi) and retrograde (Golgi-to-ER) transport, post-Golgi transport and exocytosis (Figure 2).

Cdc42 was shown to play a regulatory role in endocytic and secretory pathways, which is important for establishing and maintaining cell polarity [38]. To maintain the cell polarity, some receptors, which express on the apical or basolateral membranes, must be transported to their appropriate location after endocytosis. It has become clear that actin cytoskeleton plays a role in apical and basolateral traffic in polarized cells, and Rho GTPases regulate the actin dynamics. Thus, Rho GTPases are involved in maintaining cell polarity. In epithelial cells, Cdc42 regulates polarized endocytosis at the basolateral membrane [39]. Subsequently, Cdc42 has been found to modulate clathrin-dependent endocytosis. Inactivation of Cdc42 by microinjecting a dominant-negative mutant form (Cdc42 T17N) inhibits endocytosis in immature dendritic cells, while the constitutively activate Cdc42 mutant (Cdc42 Q61L) stimulates endocytosis in mature dendritic cells [40]. Cdc42 is responsible for the uptake of glycosyl-phosphatidylinositol-anchored proteins (GPI-APs) via a clathrin-independent endocytic pathway [41]. Cdc42 helps direct these lipid anchored proteins into the GPI-AP-enriched early endosomal compartment (GEEC). This process is governed by cholesterol-sensitive Cdc42-based actin polymerization [42,43]. During endocytosis, Cdc42 interacts with its effector, the Wiskott-Aldrich syndrome protein (N-WASP). N-WASP can then bind and activate the actin-related protein 2/3 (Arp2/3) complex, leading to actin polymerization [44]. Clathrin-mediated endocytosis involves another Cdc42 effector, called transducer of Cdc42-dependent actin assembly 1 (Toca-1). Toca-1 has three domains, an F-BAR domain, a Cdc42 binding site and a Src homology 3 (SH3) domain. The Cdc42 binding site contributes to the interaction of Toca-1 and Cdc42 by facilitating the proper folding of Toca-1, while the SH3 domain binds to N-WASP [15]. Forster resonance energy transfer (FRET) experiments have shown that Toca-1, Cdc42 and N-WASP form a trimeric complex on membrane tubules and vesicles. Cdc42 facilitates the assembly of the Toca-1/N-WASP complex by interacting with N-WASP. An important role for this complex may be to induce the formation of membrane tubules and vesicles [45].

Cdc42 has been found to affect several distinct steps in intracellular trafficking. The GTPase-defective mutant (Cdc42 Q61L) and the dominant negative mutant (Cdc42 T17N) both block the transport of glycoprotein of vesicular stomatitis virus (VSV-G) from the ER to the Golgi apparatus. By contrast, the Cdc42 F28L mutant, which binds GTP, accelerates the transport of VSV-G from the ER into the Golgi [46]. In addition, several studies have demonstrated functions for Cdc42 in retrograde trafficking through the secretory pathway. For instance, overexpression and activation of Cdc42 inhibit the retrograde transport of Shiga toxin from the Golgi apparatus to the ER [47]. Furthermore, activation of Cdc42 or knockdown of ARHGAP21 suppresses the retrograde transport of Shiga toxin from the cell periphery to the juxtanuclear Golgi region [48]. Activated Cdc42 also regulates export of bone-specific proteins from the Golgi complex to the cell surface in osteoblasts via the GEF faciogenital dysplasia protein (FGD1) [49], suggesting that Cdc42 also exerts functions in post-Golgi trafficking.

Intracellular membrane trafficking requires the actin cytoskeleton and molecular motor-dependent motility. Because Cdc42 plays a central role in regulating the actin cytoskeleton dynamics, it is possible that Cdc42 exerts its roles in intracellular trafficking through N-WASP/Arp2/3-regulated actin dynamics [50]. Cdc42 predominantly locates in the Golgi complex in mammalian cells, and it can specifically bind to ARF1-dependent vesicle-coat protein complex coatomer [46,51–53]. The coatomer/Cdc42 complex recruits N-WASP and Arp2/3 to the Golgi membrane. Importantly, p23 (a COOH-terminal dilysine motif on the putative cargo receptor) competes with Cdc42 for binding to γCOP. Once the interaction between Cdc42 and COPI is disrupted by p23, dynein is recruited to Golgi membranes to promote dynein-dependent vesicle transport. This implies that Cdc42 may help coordinate actin and microtubule-dependent motility events in the secretory pathway.

Interestingly, the signal pathway of Cdc42/N-WASP/Arp2/3 facilitates protein export by inducing production of actin filaments at the site of exocytosis. A constitutively active Cdc42 mutant can increase exocytosis in chromaffin and PC12 cells [54]. The neuronal guanine nucleotide exchange factor (intersectin-1L), one isoform of the intersectin-1 protein, enhances secretagogue-induced activation of Cdc42. Activated Cdc42 recruits N-WASP to the subplasmalemmal region to activate Arp2/3. The increased actin polymerization helps bring granules to the docking sites on the plasma membrane [55].

### 2.2. Rac1: Involvement in Endocytic and Exocytic Transport

Rac1, Ras-related C3 botulinum toxin substrate 1, is the best-characterized member of the Rac subfamily (including Rac1, Rac2, Rac3 and RhoG). Previous studies found that Rac1 regulates a diverse array of cellular events, including the formation of lamellipodia and membrane ruffles, cell cycle, cell adhesion and mobility [21]. In addition, Rac1 regulates endocytic and exocytic trafficking pathways [19] (Figure 2). Unlike Cdc42, Rac1 has not been characterized to participate in the early secretory pathway, nor has it been shown to regulate membrane trafficking at the ER/Golgi interface. Consistent with its function, Rac1 is not located in the Golgi complex [56].

Rac1 has played a characterized role in clathrin-dependent endocytosis [57]. Activation of Rac1, when expressed in intact cells, blocks transferrin-receptor-mediated endocytosis. One mechanism through which Rac1 regulates clathrin-mediated endocytosis is by modulating phosphatidylinositol-3 kinase (PI3K) and synaptojanin 2 (a polyphosphoinositide phosphatase). PI3K, a member of the phosphatidylinositol 4-phosphate 5-kinase family, is an upstream regulator of Rac1. PIP2 is phosphorylated by PI3K to generate PIP3, which induces the activation of Rac1 guanine nucleotide exchange factors (GEFs) to regulate actin rearrangement and vesicular trafficking in various cell types [58]. Recently, P-Rex1, the PI3K-dependent Rac exchange factor, has been identified as a novel regulator of the glucose transporter (GLUT4) trafficking in adipocytes. PIP3 and βγ subunits of heterotrimeric G-proteins can synergistically activate P-Rex1 [59]. P-Rex1 activates Rac1 to stimulate actin polymerization and facilitate GLUT4 transport to the plasma membrane [60]. Synaptojanin 2, a novel Rac1 effector, can interact with Rac1 directly and specifically [61]. This interaction decreases the formation of coated pits, thereby inhibiting endocytosis.

Recent studies have indicated that activated Rac1 inhibits the endocytosis of E-cadherin through the F-actin cross-linking protein, Ras GTPase-activating-like protein IQGAP1 (IQGAP1), one of its downstream targets. After being activated by trans-interacting E-cadherin, Rac1 can bind to IQGAP1, and then IQGAP1 cross-links F-actin into actin bundles, which inhibits the endocytosis of E-cadherin [62]. Moreover, Rac1 modulates ARF6-mediated clathrin-independent endocytosis through phosphatidydependentinositol-4-phosphate5-kinase (PIP5K). PIP5K binds to the polybasic region (PBR) binding domain of Rac1, whereas calmodulin (CaM) interacts with Rac1 through both the PBR and an adjacent prenyl group. Thus, CaM can disrupt the interaction between Rac1 and PIP5K through steric hindrance. Inhibition of CaM increases the Rac1/PIP5K binding interaction [63]. This interaction is essential for retaining PIP5K at the plasma membrane and for the synthesis of PIP2, which regulates actin dynamics [64].

Rac1 has also been reported to regulate calcium-dependent exocytosis in some cells. For instance, a late step of Ca^2+^-dependent neurotransmitter release is regulated by Rac1 in rat brain synaptosomes [65]. Rac1 can be associated with highly purified synaptic vesicles and is involved in the regulation of neurotransmitter release. Rac1 activation increases secretion in bovine chromaffin cells [66] and participates in cholecystokinin (CCK)-induced amylase release in pancreatic acinar cells [67]. Early studies indicated that the role of Rac1 in exocytosis is possibly relevant to forming actin-based membrane ruffles [68]. Rac1 redirects the exocytosis of recycling membranes into the sites of ruffle formation. However, the regulatory effects of Rac1 on the calcium-regulated exocytosis might rely on modulating the activity of phospholipase D1 (PLD1). PLD1 plays a major role in late steps of the exocytotic pathway [69]. As an upstream regulator of PLD1, Rac1 could strongly stimulate PLD1 activation and increase the level of phosphatidic acid (PA) at the plasma membrane [70]. The precise mechanisms of Rac1 in exocytosis will require further investigation.

### 2.3. RhoA Mainly Participates in the Regulation of Endocytic Transport

The Rho subfamily of GTPases currently consists of three members: RhoA, RhoB and RhoC. Although these isoforms are highly homologous (RhoA and RhoB share 84% amino acid sequence identity; RhoA and RhoC share 92% amino acid sequence identity [1]), they have strikingly different functions in cells. These distinct functions result from *C*-terminal sequence divergence among RhoA, RhoB and RhoC, which affects their localization. RhoA and RhoC are localized in the cytoplasm or at the plasma membrane, whereas RhoB is localized primarily on late endosomes, lysosomes and the plasma membranes. Recently, the functions of these Rho isoforms have extensively studied. RhoA has the remarkable ability to regulate actin polymerization, cell adhesion, actomyosin contractility, cellular transformation, activation of transcription and endocytosis [7,71]. RhoB plays a critical role in cytokine trafficking and cell survival, while RhoC may be able to regulate cell motility [21,72]. Here, we focus on explaining how RhoA regulates endocytosis.

RhoA helps direct endocytosis in a variety of cell types (Figure 2). For example, inactivated RhoA can reduce the uptake of oligomeric Aβ42 in N2A cells as part of the mechanism to defend cells from oligomeric Aβ42-induced neurotoxicity [73]. Activated RhoA inhibits transferrin-receptor-mediated endocytosis in Hela cells [57], the muscarinic acetylcholine receptors trafficking to the plasma membrane in HEK293 cells [74] and the uptake of fetuin-A in bovine vascular smooth muscle cells [75]. By contrast, RhoA promotes the endocytosis of interleukin 2 receptors in L_αβγ_ cells [76] and compensatory endocytosis in umbrella cells [77].

Activated RhoA may affect endocytosis by recruiting the Rho-associated coiled coil-containing protein kinases (ROCKs) to rearrange actin cytoskeleton. The actin cytoskeleton is a highly dynamic structure that promotes the invagination of apical membranes through conjugating myosin motors. As the major downstream effectors of RhoA, ROCKs are involved in two signaling pathways that reorganize the actin cytoskeleton. ROCKs inhibit the myosin light chain phosphatase (MLCP) activity by phosphorylating the myosin binding subunit of MLCP and then increasing the phosphorylation of MLC, thereby enhancing myosin II activity, stress-fiber formation and cellular contraction [78]. Moreover, the ROCK/LIM kinase (LIMK) signaling pathway inactivates cofilin, an actin depolymerization factor, resulting in the stabilization of F-actin and the increase of actomyosin contractility [79]. Suppressing the actin-filament severing activity of cofilin leads to stress-fiber formation [80].

Some upstream regulators of RhoA, including PI3K, focal adhesion kinase (FAK), GC-binding factor 2 (GCF2) and GTPase regulator associated with focal adhesion kinase-1 (GRAF1), have been reported to participate in endocytosis and membrane protein trafficking by regulating RhoA activation. Treatment with the PI3K inhibitor (LY294002) or the FAK inhibitor (PF573228) suppresses compensatory endocytosis by ~30% and ~70%, respectively, by inhibiting the activation of RhoA and then reducing the recruitment of ROCK [77]. Increasing expression of the transcription repressor, GCF2, can silence RhoA expression, leading to actin cytoskeleton disorganization. This increases the internalization of multidrug resistance-associated protein-1 (MRP1) into cytoplasm [81]. GRAF1 participates indirectly in membrane sculpting via regulating RhoA activity. The *N*-terminal BAR and PH domains of GRAF1 localize GRAF1 to membrane tubular structures, [82,83]. Thus, GRAF1 work together with RhoA in directing changes in membrane morphology during endocytosis.

Interestingly, some GAPs and GEFs affect exocytosis by regulating RhoA activity. For instance, GEF-H1, a guanine nucleotide exchange factor for RhoA, can interact with the exocyst component, Sec5. This interaction facilitates RhoA activation, which in turn influences the exocytic trafficking from the Golgi apparatus to the plasma membrane by regulating the assembly and localization of exocyst protein, Exo70 [84]. Gem-interacting protein (GMIP), a RhoA GTPase-activating protein, binds to the secretory factor, JFC1, to orchestrate actin remodeling near secretory organelles by mediating RhoA inactivation and then inducing exocytosis [85].

## 3. Rho GTPases Regulate Viral Proteins and Genome Transport in Host Cells

Given that Cdc42, Rac1 and RhoA are responsible for regulation of host cell protein trafficking, it has been proposed that they play an important role in the intracellular transport of viral proteins and genome. Our previous studies have indicated that Cdc42 and its specific GAP ARHGAP21 are both involved in the transport of influenza A virus neuraminidase (NA) protein. Expression of constitutively active Cdc42 or silencing ARHGAP21 facilitates the transport of NA to the plasma membranes, while expressing shRNA targeting Cdc42 or overexpressing ARHGAP21 significantly decreases the amount of cell surface-localized NA, according to immunofluorescence and NA activity assay [86]. Cdc42 also associates with the influenza A virus matrix protein 1 (M1), and this interaction probably contributes to virus budding [87].

In addition to influenza A virus, previous studies revealed that Cdc42, Rac1 and RhoA participate in the lifecycle of multiple viruses. For example, the endocytosis of adenovirus (Ad) and adeno-associated virus type 2 (AAV-2) involves Cdc42 and Rac1 [88–90]. The internalization of Japanese encephalitis virus (JEV) needs the help of RhoA [91]. Cdc42 and Rac1 exert influence early in herpes simplex virus type 1 (HSV-1) infection after the viruses enters the host cells [92]. RhoA is implicated in the trafficking of Kaposi’s sarcoma-associated herpes virus (KSHV) DNA to the cell nucleus [93,94]. The release of human immunodeficiency virus (HIV-1) and Marburg virus (MARV) particles from infected cells is also strongly influenced by RhoA and Cdc42, respectively [95,96].

The processes of viral endocytosis, intracellular trafficking and exocytosis in the host cells are mostly associated with Rho GTPase-regulated cytoskeletal dynamics. Treatment with CdTB (a chemical inhibitor of Rho GTPases) disrupts actin dynamics and prevents the internalization and trafficking of KSHV to the nucleus in human umbilical vein endothelial cells. Wiskostatin (an inhibitor of N-WASP) has similar effects. These suggest that the regulation of actin dynamics through the Rho GTPase/N-WASP/Arp2/3 pathway has an essential role in KSHV entry and trafficking in endothelial cells [97]. In addition, dynamic polymerization of actin in filopodia upon overexpression of Cdc42 promotes the release of MARV particles [96].

Rho GTPases are involved in the regulation of microtubule dynamics and, thus, facilitate the transport of viral pathogens in infected cells and the release of newly assembled virus progeny from host cells. It has been shown that microtubule motors were involved in regulating African swine fever virus (ASFV) entry to host cell and the trafficking of virions to the plasma membrane. Of interest, during the early stages of ASFV infection, activated Rac1 regulates microtubule dynamics by acetylating tubulin, leading to changes in ASFV intracellular transport [98]. RhoA-mDia regulated microtubule dynamics affect viral DNA trafficking in KSHV-infected cells and vaccinia virus release into the media. When infected with KSHV, the RhoA-dependent activation of Dia2 is enhanced in HFF cells [93]. Activated Dia2 directly binds to microtubules and controls their assembly dynamics. This promotes viral DNA transport to the cell nucleus [99]. Besides, dynein motors also play a role in the cytoplasmic trafficking of KSHV and the transport of its DNA to the nucleus [93]. Vaccinia virus F11L protein interacts with RhoA to inhibit the RhoA-ROCK and RhoA-mDia signaling. This is responsible for viral morphogenesis, increased peripheral microtubule dynamics and reorganization of cortical actin, thereby promoting vaccinia virus particle release from infected cells [100–102].

## 4. Cdc42, Rac1 and RhoA Contribute to Cellular Transformation and Regulate Tumor Invasion and Metastasis

Malignant cellular transformation is a complex multifaceted process. Precise spatial and temporal regulation of vesicle trafficking by Cdc42, Rac1 and RhoA is important for cell survival and function and, hence, contribute to cellular transformation. Perturbation of Cdc42, Rac1 and RhoA activity results in the loss of normal physiological function and can be related to the pathological conditions, including cellular transformation, tumor invasion and metastasis.

The relationship between Cdc42, Rac1 or RhoA and cellular transformation has been extensively studied during the past ten years. This work indicates that hyperactivation of Cdc42, Rac1 or RhoA can lead to cellular transformation. The cycle between GDP- and GTP-bound states of GTPase is critical for the transforming potential of Cdc42, Rac1 and RhoA [103,104]. The spontaneously activated (so-called “fast-cycling”) mutants of Cdc42, Rac1 and RhoA that facilitate the intrinsic nucleotide exchange rate, but still exhibit normal GTP hydrolytic activity, can mediate cellular transformation induced by the Dbl oncoprotein in NIH3T3 cells [105]. Cdc42 activation can enhance anchorage-independent cell growth by boosting filopodia formation and generating large, multinucleated cells. RhoA activation results in the loss of contact inhibition and the increase of stress fiber and focal adhesion complexes. Rac1 activation enhances the accumulation of cortical actin at the cell periphery [105].

Specific GAPs can inhibit the activation of Cdc42, Rac1 and RhoA by increasing intrinsic GTPase activity, leading to suppression of tumor formation. For example, deletion of liver cancer 1 (DLC-1), a RhoA and Cdc42-specific GAP, inhibits the growth of various types of cancer, including lung, breast, prostate, kidney, colon, uterus, ovary and stomach through altering the actin cytoskeleton [106,107]. Ankyrin repeat and pleckstrin homology (PH) domain 3 (ARAP3), a specific Rho GAP of Cdc42, Rac1 and RhoA, has been reported to inhibit peritoneal dissemination of scirrhous gastric carcinoma cells [106,108]. The functions of Rho GDIs in cellular transformation are very complex [104] and need further investigation. In some cases, Rho GDIs are thought to inhibit Rho GTPases activation and function as tumor suppressors. Knocking down of RhoGDI1 can promote cancer cell metastasis via inducing RhoA activation [109]. Clinical evidence indicates that RhoGDI2 is a metastasis suppressor in bladder cancer [110]. However, Rho GDIs may not simply inhibit tumor cell metastasis, but also be required for cellular transformation. One study has shown that the interaction between Cdc42 and RhoGDI is essential for Cdc42-mediated cellular transformation. NIH 3T3 cells expressing the Cdc42 (F28L, R66A) double mutant, which is ineffective in binding to RhoGDI, fails to proliferate in low (1%) concentrations of serum and form colonies in soft agar, suggesting that RhoGDI could be involved in cellular transformation [111].

Cdc42, Rac1 and RhoA have the ability to promote cellular transformation, not only due to their own aberrant activation, but also due to their ability to modify oncoproteins, such as Ras, EGFR and sarcoma (Src). Early studies have revealed that Ras-dependent transformation requires functional Cdc42, Rac1 and RhoA [112–115]. These Rho GTPases can impinge on Ras-induced signaling pathways. For example, Cdc42 and Rac1 activate PI3K through indirect cooperative positive feedback [116]. Fibroblast growth factor-2 (FGF-2)-induced activation of Ras/mitogen-activated protein kinase (MAPK) signaling and c-Jun *N*-terminal kinase (JNK) activation require Rac1 activity in human breast cell line MCF7 [117].The p21-activated kinases (PAKs), downstream effectors of Cdc42, bind to activated Cdc42 and Rac1 to activate various downstream signaling cascades. This regulates cancer progression by promoting cell proliferation, survival, motility and angiogenesis [118,119]. EGFR is often overexpressed or abnormally activated to activate mitogenic signaling pathways in tumor progression. Activation of Cdc42 can prevent EGFR degradation to sustain EGFR signaling and then promote cellular transformation through interacting with c-Cbl, which plays a role in the initiation of EGFR degradation [120]. In addition, Cdc42 may further regulate EGFR-mediated transformation by altering its cellular trafficking. RhoA and ROCK have been shown to negatively regulate EGFR endocytosis. The active form of ROCK can phosphorylate endophilin A1 at Thr-14 and then affect the recruitment of endophilin A1 to the EGFR-c-Cbl-CIN85 complex, leading to the reduction of EGFR endocytosis [121,122]. Similarly, Cdc42, Rac1 and RhoA play roles in cellular transformation mediated by Src [123]. For instance, mDia1, a downstream effector of Rho, is essential for v-Src-induced cellular transformation. Previous studies have shown that v-Src-induced transformation and podosome formation are inhibited in mDia1-deficient cells [124]. This can be explained by the fact that mDia1 deficiency decreases the levels of several tyrosine-phosphorylated proteins, most of which are high molecular mass proteins (100 to 250 kDa), in v-Src-transformed cells. Subsequently, the translocation of v-Src from the perinuclear region to the cell periphery is impaired, leading to the downregulation of downstream signaling pathways. This ultimately results in the suppression of v-Src-induced cell transformation [124].

Transformed cells exhibit invasive and metastatic properties and the capacity to form invadopodia at the leading edge, which is regulated by actin cytoskeleton reorganization [125]. Cdc42 is thought to be important for invadopodia formation, due to induction of actin polymerization via activating the N-WASP/Arp2/3 pathway [126]. N-WASP and the Arp2/3 complex are specifically localized at invadopodia and are essential for invadopodia formation in carcinoma cells. Treatment with N-WASP siRNA or transfection with a N-WASP dominant-negative mutant (lacking the sequence that is required for binding to the Arp2/3 complex) could reduce invadopodia formation in MTLn3 cells [126]. The precise function of N-WASP in invadopodia formation may be related to its ability to promote the assembly of actin filaments through interacting with the Arp2/3 complex or increasing the internalization of degraded matrix components and the recycling of the invadopodia components [127]. In addition, it has been shown that the presence of Rac1 inhibitor, NSC23766, blocks invadopodia formation and matrix degradation [128]. The downstream effector of Rac1, WAVE2, is an important regulator of melanoma invasion and is involved in metastasis. Both enhanced Rac1 activation and increased WAVE2 expression synergistically contribute to B16F10 cell invasion [129]. Moreover, RhoA can also promote tumor cell invasion through regulating invadopodia formation. Inhibition of RhoA activity with C3 or transfection with dominant-negative RhoA mutant disrupts F-actin accumulation and invadopodia formation [130]. The RhoA effectors, ROCKs and formins, both also have an effect on invadopodia formation. Inhibition of ROCK with specific siRNA reduces invadopodia and decreases tumor cell invasion and knockdown of Dia1, −2 or −3 can inhibit the formation of invadopodia and invasion capacity of MDA-MB-231 cells [131,132]. Taken together, these data suggest that Cdc42, Rac1 and RhoA are all closely associated with the invasion and metastasis of tumor cells through regulating the formation of invadopodia.

## 5. Conclusions

**Figure 3:**
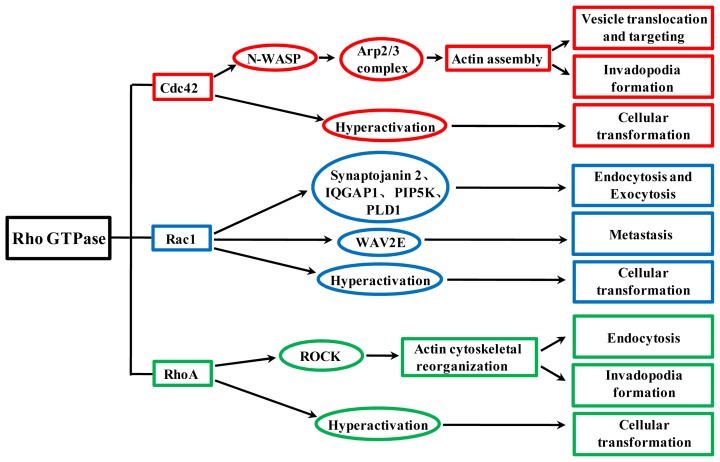
Involvement of Cdc42, Rac1 and RhoA in vesicle trafficking, cellular transformation, invadopodia formation and metastasis. Cdc42 regulates vesicle trafficking and invadopodia formation through Wiskott-Aldrich syndrome protein (N-WASP)-mediated Arp2/3 signaling pathway. Rac1 has an effect on endocytosis and exocytosis via acting on its downstream effectors, including synaptojanin 2, IQGAP1, PIP5K and PLD1, and is involved in metastasis through its downstream molecule, WAVE2. RhoA activates its major downstream effector, Rho-associated coiled coil-containing protein kinases (ROCK), to modulate endocytosis and invadopodia formation through affecting actin dynamics. Hyperactivation of Cdc42, Rac1 or RhoA can result in cellular transformation.

Because Rho GTPases intimately participate in the regulation of actin cytoskeleton, they play key roles in various physiological and pathological processes, including intracellular transport and tumor progression from transformation to metastasis (Figure 3).

Extensive studies have revealed the pleiotropic effects of Cdc42, Rac1 and RhoA on vesicle trafficking. Although each of them has a specific role, parts of their functions are often overlapping, such as regulating endocytosis. Cdc42 promotes endocytosis through regulation of the Cdc42/Toca-1/N-WASP/Arp2/3 signaling pathway. Rac1 functions in endocytosis by controlling PI3K, synaptojanin 2, IQGAP1 or being involved in Arf6-dependent endocytosis. ROCK plays a crucial role in the RhoA-induced endocytosis. Increasing evidence suggests that there may exist cross talk between the Rho GTPases. However, the precise mechanisms by which signals from these different Rho GTPases are coordinated remain unknown.

In light of previous work, one can conclude that there are cross effects between vesicle trafficking and cellular transformation mediated by Cdc42, Rac1 and RhoA. The fact that the two cellular events share the same effectors might explain the phenomenon described above. For example, as a target of Cdc42, COPI can regulate Cdc42-mediated vesicle trafficking and cellular transformation simultaneously [46,51]. Cdc42 mutants that are unable to bind γCOP affect both trafficking of VSV-G from the ER to the Golgi apparatus and cellular transformation [46]. The role of ROCK in regulating RhoA-induced endocytosis and cellular transformation has also been documented. Treatment with the ROCK inhibitor, Y-27632, could reduce actin stress fibers, disrupt endocytosis and impair the transforming activity of RhoA. However, how Rac1 effectors play roles in both vesicle trafficking and cellular transformation still needs to be determined.

This review focuses mostly on Rho family hallmarks members (Cdc42, Rac1 and RhoA), but some of other members are also closely associated with intracellular transport and tumorigenesis. In the future, more precise investigation of the regulatory roles of the “classical” and “nonclassical” members of the Rho GTPases family will yield additional fascinating insights.
